# Deceptive *Cypripedium calceolus* shares more floral scent compounds with co-flowering rewarding species than those species share among each other

**DOI:** 10.3389/fpls.2025.1627890

**Published:** 2025-08-12

**Authors:** Corinna Etl, Florian Etl, Robin Guilhot, Herbert Braunschmid, Karin Gross, Stefan Dötterl

**Affiliations:** ^1^ Department of Environment and Biodiversity, Paris Lodron University of Salzburg, Salzburg, Austria; ^2^ Department of Botany and Biodiversity Research, University of Vienna, Vienna, Austria; ^3^ CHITINE – Etudes Entomologiques, Caluire-et-Cuire, France

**Keywords:** volatile organic compounds, deceptive pollination, co-flowering community, generalized food-deception, mimicry, orchid

## Abstract

The vast majority of flowering plants depend on animal pollinators for sexual reproduction. These plants usually provide a reward, such as nectar and/or pollen, to their pollinators, and floral scent is often key to attract them. Some plants, however, do not provide any such reward, though they advertise one. Even though it is well known that such a food-deceptive pollination strategy is particularly common in orchids, the role of floral scent in attracting pollinators in such systems is often poorly understood. In this study, we compared the floral scent of the Eurasian deceptive lady’s slipper orchid *Cypripedium calceolus* with six co-flowering rewarding species visited by the same pollinators. *Cypripedium calceolus* produced more floral scent compounds than the co-flowering rewarding species together and differed in the floral scent composition from them. However, *C. calceolus* shared at least one compound with each co-flowering rewarding species, including widespread and less widespread compounds among flower scents, and had more compounds in common with the co-flowering rewarding species than they had with each other. Several compounds of *C. calceolus*, such as the aliphatic compounds 1-octanol, octyl acetate, and decyl acetate, did not occur in co-flowering plants but are known as pheromones of pollinating bees. Together, our results suggest that *C. calceolus* not only emits compounds that are generally common among flowering plants and attractive to many pollinators but specifically imitates floral scent compounds of multiple co-flowering plant species/pheromones of bees. These findings provide valuable insights into the ecology and evolution of floral scent in deceptive pollination systems in orchids.

## 1 Introduction

The vast majority of angiosperms rely on animal pollinators, primarily insects, for sexual reproduction (Ollerton et al., 2011). Most of these plants provide rewards, such as pollen and nectar (Endress, 1996), which they typically advertise through visual and olfactory cues (Chittka and Raine, 2006). However, not all plant species that signal a reward actually provide one (Johnson and Schiestl, 2016). Such deceptive pollination systems are particularly common in orchids (Orchidaceae), where approximately a third of all species studied do not produce floral rewards (van der Pijl and Dodson, 1966; Dafni, 1984; Nilsson, 1992). Most of the deceptive orchids are food deceptive (60% of the deceptive species) or sexually deceptive (38%) and exploit the food- or mate-seeking behavior, respectively, of their pollinators (Ackerman et al., 2023). In sexually deceptive orchids, the mechanisms of deception by highly specific floral shapes, structures, and especially scents reminiscent of relevant traits of female insects are well studied (Johnson and Schiestl, 2016; Peakall, 2023; Slavković and Bendahmane, 2025), whereas in food-deceptive systems, there are large gaps in the understanding of which floral traits shape these interactions (Johnson and Schiestl, 2016).

There are two main strategies in food-deceptive orchids (Jersáková et al., 2009; Schiestl and Schlüter, 2009). One is “Batesian floral mimicry” in which the deceptive species mimics a specific model species in one or more floral traits to attract the nectar- or pollen-seeking pollinators of the model species such as bees, beetles, and flies (Dafni, 1984; Roy and Widmer, 1999; van der Cingel, 2001). Previous studies have highlighted the importance of visual similarity between models and mimics in explaining the evolutionary drivers of specialized food mimicry (Peter and Johnson, 2008; Jersáková et al., 2012). For example, in the South African orchid *Disia pulchra*, which is pollinated by long-proboscid tabanid flies, artificial flowers with the same color spectra as the model species, but without scent, successfully attracted the pollinators (Jersáková et al., 2012). The role of floral scent in such Batesian mimicry systems seems to be less important (but see Scaccabarozzi et al., 2025), at least in short-distance attraction. However, it has been hypothesized that floral scent plays a more important role in long-distance attraction in systems such as between the orchid *Orchis israelitica* and its model, the lily *Bellevalia flexuosa* (Galizia et al., 2005). In the other, more common food-deceptive strategy, which is “generalized food deception”, pollinators are most likely deceived by floral traits widespread in rewarding flowers (Johnson and Schiestl, 2016). As an intermediate between Batesian floral mimicry and generalized food deception, guild mimicry has been suggested, in which the deceptive species imitates guilds of co-flowering species. Such guild mimicry has recently been suggested for *Traunsteinera globosa* (Jersáková et al., 2016).

A plant species with a yet unknown deceptive pollination strategy is the charismatic and widespread Eurasian lady slipper orchid *Cypripedium calceolus*. It has a bright yellow color and some widespread floral scent compounds, such as linalool and benzaldehyde (Nilsson, 1979; Braunschmid et al., 2017), that point to a generalized food-deceptive strategy. However, the flowers also release compounds less widespread among floral scents, such as (*Z*)-3-nonenyl acetate and lilac alcohol (Braunschmid et al., 2017), and even compounds described as pheromones of some of its pollinators, such as decyl acetate (Tengö and Bergström, 1977; Nilsson, 1979), rather indicating mimicry of specific plants and/or insects. The compounds attractive to the pollinators (mainly bees, but also hoverflies) (Braunschmid et al., 2021) need, however, to be determined. Also, the floral scents of co-flowering rewarding species of *C. calceolus* remain to be characterized, and the extent to which the floral scent of *C. calceolus* resembles the scents of these plants remains to be assessed (Schiestl, 2005; Jersáková et al., 2006).

In this study, we assessed the similarity of the floral scent of *C. calceolus* to co-flowering rewarding plant species in a population in the Bavarian Alps, Germany. Specifically, we asked (1) which floral scent compounds were shared between *C. calceolus* and each of the co-flowering plant species as well as which compounds the co-flowering plant species shared among each other and (2) how similar they were in their relative scent composition. If *C. calceolus* shared compounds generally widespread among floral scents with its co-flowering rewarding plant species, it would point to generalized food deception. If, however, it imitated the scent of specific co-flowering rewarding species, mimicry would more likely be involved.

## 2 Materials and methods

### 2.1 Study species

*Cypripedium calceolus* L. is a terrestrial, perennial orchid. Its distribution range is the boreal and temperate zones of Europe and Asia, and it grows in a variety of habitats, such as open to medium-shaded deciduous and coniferous forests, and alpine meadows and rubble, but predominantly on calcareous soil (Cribb, 1997). It flowers from May to July (in May/June in our focal population). *Cypripedium calceolus* has a plant height of up to 60 cm and the largest and most conspicuous flowers among European orchids. The inflorescence consists of one to two, rarely more, flowers. The yellow, 3- to 4-cm-long shoe-shaped labellum acts as a semi-trap. Once trapped, the pollinators can only escape through a posterior opening where they come into contact with reproductive organs (Cribb, 1997; Kull, 1999; Braunschmid et al., 2017). The remaining two petals and the three sepals have a lanceolate shape and a purple-brown color. The pollen grains are aggregated in a sticky smear. The flowers produce an apple- or apricot-like scent. Previous studies have shown that floral scent differs among regions and populations (Braunschmid et al., 2017, 2021). *Cypripedium calceolus* can reproduce vegetatively via horizontal rhizomes, is self-compatible, but relies on small insect pollinators for successful pollination (Nilsson, 1979 and references therein). The primary pollinators are various solitary bees, such as *Lasioglossum* spp. (*L. bavaricum*, the *L. calceatum*/*L. albipes* species complex, *L. fratellum*, *L. fulvicorne*, *L. leucozonium*, *L. morio*, *L. quadrinotatum*) and *Andrena* spp. (*A. bicolor*, *A. cineraria*, *A. fucata*, *A. haemorrhoa*, *A. helvola*, *A. jacobi* [=*A. carantonica*], *A. nigroaenea*, *A. praecox*, *A. tibialis*), and also hoverflies (*Eristalis rupium*, *Pipiza austriaca*, *Platycheirus albimanus*) (Nilsson, 1979; Braunschmid et al., 2017), which are most likely attracted by a combination of visual and olfactory cues (Daumann, 1968; Nilsson, 1979; Bergström et al., 1992; Braunschmid et al., 2017). Capsules contain several thousands of dust seeds that are wind dispersed (Kull, 1999).

### 2.2 Study site

The study was carried out in a *C. calceolus* population on the shore of the mountain lake Königssee, Berchtesgaden National Park, Bavaria, Germany. It is one of the populations with the highest number of individuals (1,000–2,000 shoots) and the population with the highest number of scent compounds and the highest scent emission of the four populations included in the study by Braunschmid et al. (2017). At this site, *C. calceolus* grows in a wet grassland patch near the lake shore, surrounded by very light forests of *Salix* spp. and *Picea abies*, and is mainly pollinated by the *Lasioglossum calceatum*/*L. albipes* species complex but also other solitary bees and some hoverflies (Braunschmid et al., 2017).

### 2.3 Assessment of co-flowering plant community

**Figure 1:**
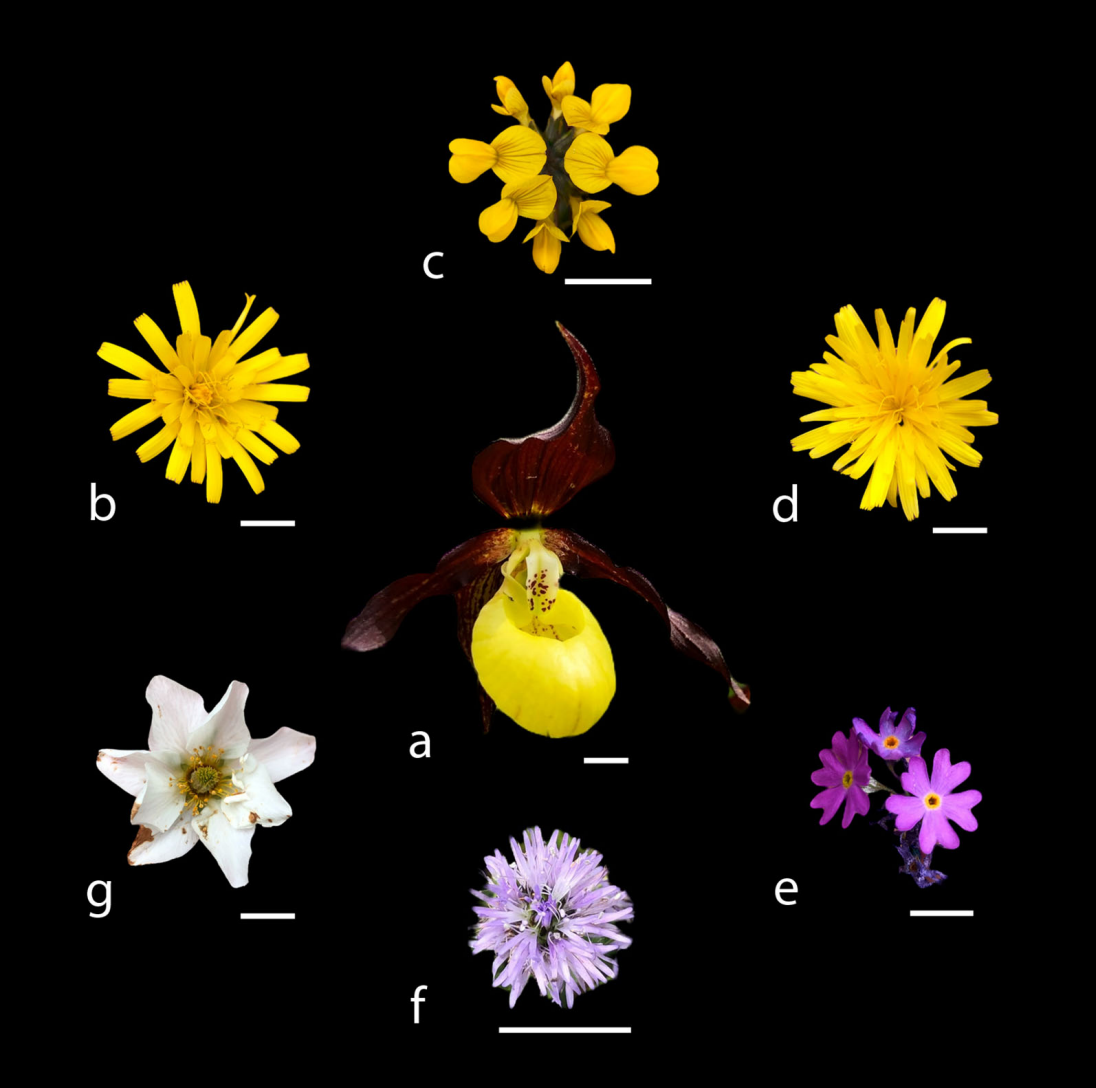
Photos of the pollination units of *Cypripedium calceolus* **(a)** and the six co-flowering rewarding species that emitted any detectable scent and were included in the statistical analyses: *Hieracium bifidum* **(b)**, *Hippocrepis comosa* **(c)**, *Leontodon incanus* **(d)**, *Primula farinosa* **(e)**, *Globularia cordifolia* **(f)**, and *Dryas octopetala* **(g)**. White bars next to each pollination unit represent 1 cm.

The co-flowering rewarding community was assessed and defined as all species in the vicinity (radius of 10 m) of flowering *C. calceolus* individuals that are known (e.g., Müller, 1881; Westrich, 2019; preliminary own observations) to be visited by insects (species, co-generics) that have been observed as flower visitors/pollinators of *C. calceolus* (Braunschmid et al., 2017, 2021). This resulted in eight species belonging to five families: *Leontodon incanus* L. (pollinators: e.g., *Halictus rubicundus*, *H. tumulorum*, the *Lasioglossum calceatum/L. albipes* species complex, *L. leucozonium*), *Hieracium bifidum* Kit. ex Hornem. (e.g., the *Lasioglossum calceatum*/*L. albipes* species complex), and *Bellidiastrum michelii* Cass. (e.g., *Halictus* sp., *Nomada* sp.) of the Asteraceae; *Dryas octopetala* L. (e.g., *Lasioglossum albipes*, *L. morio*) and *Potentilla erecta* (L.) Raeusch (*Lasioglossum* sp.) of the Rosaceae; *Hippocrepis comosa* L. (*Lasioglossum calceatum*) of the Fabaceae; *Globularia cordifolia* L. (*Halictus* sp. and/or *Lasioglossum* sp.) of the Plantaginaceae; and *Primula farinosa* L. (*Halictus* sp. and/or *Lasioglossum* sp.) of the Primulaceae (**Figure 1**).

### 2.4 Floral scent collection and analysis

The scent data on 14 individuals of *C. calceolus* of the study population were from Braunschmid et al. (2017), who collected floral scent from one flower per individual in May 2014 using dynamic headspace. We collected the floral scent of the eight co-flowering rewarding species using the same approach as Braunschmid et al. (2017). All our sampling took place, as for *C. calceolus* (Braunschmid et al., 2017), between 10:00 and 16:00. Floral scent was collected from five individuals each of *L. incanus*, *D. octopetala*, *H. comosa*, *G. cordifolia*, *P. farinosa*, and *P. erecta*, from seven individuals of *H. bifidum*, and from three individuals of *B. michelii*. Samples were collected in 2023 for all individuals except for two *H. bifidum* individuals and three *G. cordifolia* individuals, which were collected in 2015. For scent collection, one inflorescence (five inflorescences for *P. erecta*) was bagged with a polyethylene oven bag (10 × 30 cm; Toppits, Germany), and the scent was trapped for 30 min (5 min in case of *P. farinosa*) directly after bagging on an adsorbent tube (quartz glass tube: length 25 mm; inner diameter 2 mm) filled with 1.5 mg each of Carbotrap B (mesh 20–40, Supelco, Germany) and Tenax TA (mesh 60–80; Supelco, Germany). For scent collection, a rotary vane pump G 12/01 EB (Gardner Denver, Germany) with a flow of 200 ml/min was used. Negative controls were obtained by conducting the same procedure but for empty oven bags (*n* = 3) and two leaf samples per species.

Samples were stored in a freezer (−20°C) and analyzed within 2 weeks after collection with a gas chromatograph coupled to a mass spectrometer (GC/MS-QP2010 Ultra, Shimadzu Corporation, Japan), and to a thermal desorption (TD) unit (TD-20, Shimadzu, Japan), and equipped with a ZB-5 fused silica column (5% phenyl polydimethylsiloxane; 60-m long, inner diameter 0.25 mm, film thickness 0.25 μm, Phenomenex, USA), which was the same setup used by Braunschmid et al. (2017). Using the same settings as Braunschmid et al. (2017), samples were desorbed at 250°C for 15 min (flow: 25 ml/min) and cryofocused on a cold trap at −20°C in the TD-20, before they were transferred to the GC (cold trap heated to 250°C, transfer line from TD-20 to GC set to 260°C). Samples were run at a column flow (carrier gas: helium) of 1.5 ml/min. GC oven temperature started at 40°C, then increased by 6°C per min to 250°C, and was held for 1 min. The MS interface was set at 260°C and the ion source at 200°C. Mass spectra were taken at 70 eV (in EI mode) from m/z 30 to 350. The GC/MS data were processed using GCMSolution Version 4.11 software (Shimadzu Corporation, Japan). Compounds were tentatively identified by matches with the NIST 11, Wiley 9, FFNSC 2, Essential Oils, and Robert P. Adams 2007 mass spectral and retention index data bases and were confirmed by comparing mass spectra and retention times with those of authentic standards available in the stock collections of the Plant Ecology lab at the University of Salzburg. Known amounts of monoterpenes, aliphatics, and aromatics were injected into the GC/MS system, and mean peak areas of these compounds were used to calculate the total absolute amount of scent in our samples (see Dötterl et al., 2005). For analysis, we then calculated the scent emitted per pollination unit per hour. The pollination unit was an inflorescence for all co-flowering species except for *D. octopetala*, in which it was a single flower.

In all samples of *P. erecta* and *B. michelii* as well as in one sample of *L. incanus*, no flower-specific scent compounds were detected. Thus, *P. erecta* and *B. michelii* and the scentless sample of *L. incanus* were excluded from all statistical analyses and visualizations.

### 2.5 Statistical analyses

Data were processed and visualized using the statistical software program R (version 4.0.3; R Core Team, 2020) unless described otherwise.

We analyzed differences in the number of compounds emitted in two ways. At the level of individuals, we run a Kruskal–Wallis rank sum test in the *base* package in R followed by a Dunn’s test of multiple comparisons in the R package *FSA* (Ogle et al., 2021) with species as factor to assess whether the number of compounds emitted per sample differed among species.

We visualized shared and non-shared floral scent compounds between *C. calceolus* and the co-flowering species by generating a chord diagram based on binary data (presence/absence) using the R package *circlize* (Gu et al., 2014). Differences in the number of compounds shared between *C. calceolus* and the rewarding co-flowering plants and among the co-flowering plants were analyzed with a Mann–Whitney U-test in the *base* package in R.

Differences in relative amounts of each scent compound were visualized with non-metric multidimensional scaling (NMDS) based on the Bray–Curtis similarities of relative amounts of each scent compound using the *vegan* (Oksanen et al., 2020) and *ggplot2* (Wickham, 2016) R packages and statistically analyzed using a permutational multivariate analysis of variance (PERMANOVA) in PRIMER 6.1.15 (Clarke and Gogley, 2006) with PERMANOVA+ for PRIMER 1.0.5 (Anderson et al., 2008) based on Bray–Curtis similarities, with species as fixed factor, and using 9,999 permutations. Differences among species in multivariate dispersion in scent composition were assessed using a permutational analysis of multivariate dispersion (PERMDISP) in PRIMER 6.1.15 (Clarke and Gogley, 2006) with PERMANOVA+ for PRIMER 1.0.5 (Anderson et al., 2008), again based on Bray–Curtis similarities, species as factor, and 9,999 permutations.

## 3 Results

The total amount of scent emitted per pollination unit varied among species (**Table 1**; **Supplementary Table S1**). With a mean emission rate of 156 ng/h, *C. calceolus* had an intermediate scent emission compared to that of the six co-flowering species. It was in the same order of magnitude as the mean scent emission rate of *H. bifidum* and *P. farinosa*, whereas the mean scent emission rate was almost 10 times higher in *H. comosa* and less than half the amount in *D. octopetala*, *L. incanus*, and *G. cordifolia* (**Table 1**).

**Table 1:** Sample size (# individuals), number of compounds across all samples of a species (# compounds), and mean (minimum–maximum) total amout of scent trapped (ng/h per pollination unit) and relative amount (%) of floral scent compounds of *Cypripedium calceolus* and the six co-flowering rewarding species *Hieracium bifidum, Leontodon incanus, Globularia cordifolia, Primula farinosa, Dryas octopetala*, and *Hippocrepis comosa*.

RI	Trait	*Hieracium bifidum*	*Leontodon incanus*	*Globularia cordifolia*	*Primula farinosa*	*Dryas octopetala*	*Hippocrepis comosa*	*Cypripedium calceolus*
	# individuals	7	4	5	5	5	5	14
	# compounds	14	2	3	9	11	23	67
	Total amount of scent trapped (ng/h per pollination unit)	187 (52–544)	22 (14–31)	62 (14–124)	221 (31–672)	35 (1–81)	1,533 (224–2,191)	156 (34–652)
Relative amounts (%)								
Aliphatic compounds								
855	(*Z*)-3-Hexen-1-ol*	–	–	–	–	–	–	1 (0–4)
866	1-Hexanol*	–	–	–	–	–	–	tr (tr–1)
902	Heptanal*	–	–	–	–	–	–	2 (tr–10)
913	Pentyl acetate	–	–	–	–	–	–	tr (0–1)
1,006	(*Z*)-3-Hexenyl acetate*	–	–	–	–	–	–	3 (1–11)
1,011	Hexyl acetate*	–	–	–	–	–	–	4 (2–9)
1,070	1-Octanol*	–	–	–	–	–	–	1 (tr–3)
1,111	Heptyl acetate*	–	–	–	–	–	–	1 (1–2)
1,122	3-Octyl acetate	–	–	–	–	–	–	tr (0–tr)
1,129	Octyl formate	–	–	–	–	–	–	tr (0–tr)
1,162	Octanoic acid*	–	–	–	–	–	–	tr (0–1)
1,200	(*Z*)- or (*E*)-2-Octenyl acetate	–	–	–	–	–	–	tr (0–tr)
1,210	Octyl acetate*	–	–	–	–	–	–	**32 (1–46)**
1,272	1-Decanol*	–	–	–	–	–	–	tr (0–1)
1,295	(*Z*)-3-Nonenyl acetate	–	–	–	–	–	–	tr (tr–1)
1,309	Nonyl acetate	–	–	–	–	–	–	tr (tr–2)
1,409	Decyl acetate*	–	–	–	–	–	–	**8 (tr–17)**
1,475	1-Dodecanol*	–	–	–	–	–	–	tr (0–2)
1,608	Dodecyl acetate	–	–	–	–	–	–	tr (0–1)
1,808	Tetradecyl acetate*	–	–	–	–	–	–	tr (0–tr)
RI	Trait	*Hieracium bifidum*	*Leontodon incanus*	*Globularia cordifolia*	*Primula farinosa*	*Dryas octopetala*	*Hippocrepis comosa*	*Cypripedium calceolus*
Aromatic compounds								
966	Benzaldehyde*	**58 (34–100)**	**69 (0–100)**	–	–	–	–	4 (tr–29)
1,025	*p*-Methylanisole*	–	–	–	tr (0–1)	–	–	tr (0–tr)
1,037	Benzyl alcohol*	**25 (0–64)**	**31 (0–100)**	–	**16 (0–46)**	–	–	1 (0–6)
1,048	Phenylacetaldehyde*	5 (0–15)	–	–	–	–	–	tr (0–tr)
1,074	*p*-Cresol*	–	–	–	**18 (0–48)**	–	–	tr (0–2)
1,082	Benzyl formate	–	–	–	–	–	–	tr (0–tr)
1,095	Guaiacol*	–	–	5 (1–16)	–	–	–	–
1,120	2-Phenylethanol*	1 (0–2)	–	–	–	–	–	tr (0–1)
1,148	1,2-Dimethoxybenzene*	–	–	**77 (57–96)**	–	–	–	–
1,168	Benzyl acetate*	–	–	–	–	–	–	tr (0–2)
1,188	*p*-Creosol	–	–	–	1 (0–2)	–	–	–
1,205	Methyl salicylate*	–	–	–	–	–	1 (0–2)	tr (0–1)
1,262	2-Phenylethyl acetate*	–	–	–	–	–	–	1 (0–3)
1,366	Eugenol*	tr (0–tr)	–	**17 (3–37)**	–	–	–	tr (0–tr)
1,385	Methyl-2-hydroxy-3-phenylpropionate*	–	–	–	–	–	**27 (21–34)**	–
1,669	*cf*. 1,4-Dimethylindanyl acetate	–	–	–	–	–	–	tr (0–2)
Terpenoids								
987	6-Methyl-5-hepten-2-one*	–	–	–	–	–	–	2 (0–3)
993	β-Myrcene*	–	–	–	–	–	–	tr (0–1)
1,018	Pinocarvone*	–	–	–	–	–	–	tr (0–tr)
1,039	(*Z*)-β-Ocimene*	–	–	–	–	–	tr (0–1)	tr (0–tr)
1,045	Lavender lactone*	–	–	–	–	**24 (2–100)**	–	tr (0–1)
1,050	(*E*)-β-Ocimene*	–	–	–	–	–	**8 (7–9)**	tr (0–1)
1,056	(*Z*)-Arbusculone	–	–	–	–	**9 (0–20)**	–	–
1,074	(*E*)-Arbusculone	–	–	–	–	5 (0–16)	–	–
1,078	(*Z*)-Linalool oxide furanoid*	–	–	–	–	–	–	tr (0–tr)
1,094	(*E*)-Linalool oxide furanoid*	tr (0–1)	–	–	–	–	–	1 (tr–2)
1,103	Linalool*	–	–	–	–	–	1 (0–2)	**30 (14–63)**
1,132	*allo*-Ocimene*	–	–	–	–	–	tr (tr–tr)	tr (0–tr)
1,137	Epoxyoxoisophorone*	–	–	–	1 (tr–1)	–	–	tr (0–tr)
1,140	*neoallo*-Ocimene*	–	–	–	–	–	tr (0–tr)	–
1,144	(*E*)-Ocimene epoxide*	–	–	–	–	–	tr (tr–tr)	–
1,148	Lilac aldehyde A*	–	–	–	–	**13 (0–20)**	–	–
1,150	4-Oxoisophorone*	–	–	–	**60 (24–97)**	–	–	2 (0–5)
1,157	Lilac aldehyde B+C*	–	–	–	–	**32 (0–58)**	–	–
1,172	Lilac aldehyde D*	–	–	–	–	**8 (0–15)**	–	–
1,173	Dihydrooxoisophorone	–	–	–	1 (0–4)	–	–	–
1,176	(*Z*)-Linalool oxide pyranoid*	–	–	–	–	–	–	tr (0–tr)
1,180	(*E*)-Linalool oxide pyranoid*	1 (0–2)	–	–	–	–	–	tr (tr–tr)
1,216	4-Methyleneisophorone	–	–	–	2 (tr–5)	–	–	–
1,219	Lilac alcohol B+C*	–	–	–	–	2 (0–3)	–	tr (0–tr)
1,233	Lilac alcohol D*	–	–	–	–	1 (0–2)	–	tr (0–tr)
1,233	Nerol*	–	–	–	–	–	–	tr (0–tr)
1,257	Geraniol*	–	–	–	–	–	–	tr (0–tr)
1,292	(*E*)-Linalool oxide acetate pyranoid	–	–	–	–	–	–	tr (0–tr)
1,348–1,363	Lilac alcohol formate A-D	–	–	–	–	–	–	tr (0–1)
1,349	8-Oxolinalool	–	–	–	–	4 (0–7)	–	–
1,384	Geranyl acetate*	–	–	–	–	–	–	tr (0–tr)
1,395	α-Copaene*	2 (0–7)	–	–	–	–	–	–
1,407	β-Isocomene	tr (0–1)	–	–	–	–	–	–
1,444	β-Caryophyllene*	–	–	–	–	–	tr (0–tr)	–
1,462	(*E*)-β-Farnesene*	–	–	–	–	–	–	tr (0–tr)
1,498	(*Z*,*E*)-α-Farnesene	–	–	–	–	–	–	tr (0–tr)
1,513	(*E*,*E*)-α-Farnesene*	–	–	–	–	–	–	tr (0–1)
C5-branched chain compounds								
876	Isoamyl acetate*	–	–	–	–	–	–	tr (0–tr)
Nitrogen-containing compounds								
1,228	2-Aminobenzaldehyde*	–	–	–	–	–	2 (2–3)	–
1,305	Indole*	–	–	–	–	–	**18 (13–27)**	tr (0–tr)
1,422	N-Formyl-2-aminobenzaldehyde	–	–	–	–	–	**12 (4–23)**	–
Miscellaneous cyclic compounds								
1,390	(*E*)-Jasmone	–	–	–	–	–	tr (0–tr)	–
1,415	(*Z*)-Jasmone*	–	–	–	–	–	4 (2–6)	–
Unknown compounds								
1,295	*m/z*: 106, 135, 77, 79, 107	–	–	–	–	–	23 (10–43)	–
	Other unknown compounds pooled^(25)^	7 (0–30)^5^	–	–	tr (0–1)^1^	3 (0–5)^2^	3 (1–6)^8^	tr (0–1)^9^

The compounds are sorted by compound class and retention index (RI). The number of individuals, the number of compounds emitted, and the total absolute amount are also given. Values >5% are highlighted in bold. The superscribed numbers for the "other unknown compounds pooled" give the number of compounds that were pooled across all species and separately for each species.

*Compound identification verified through authentic standard; tr, values <0.5% but >0%; - compound not detected.

**Figure 2:**
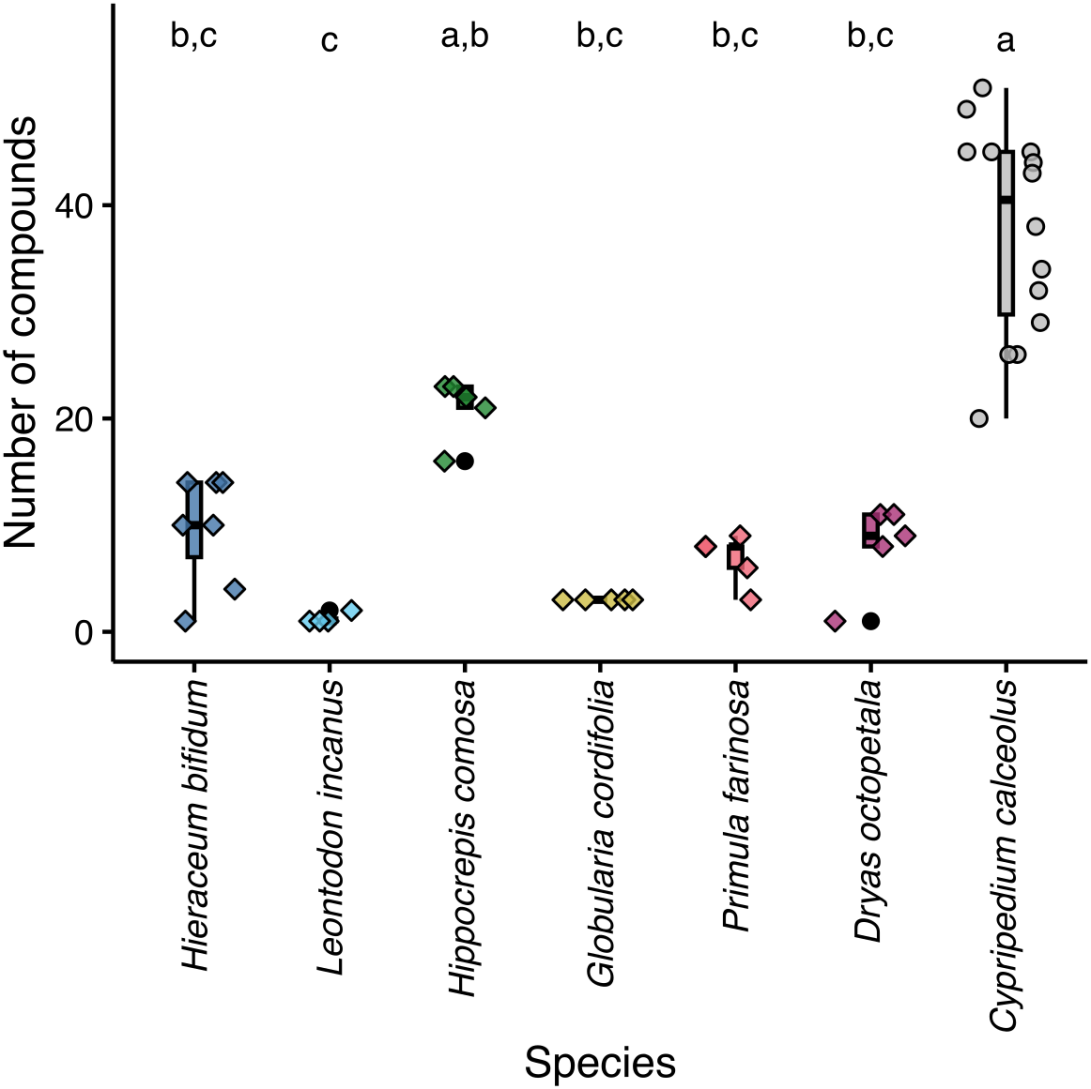
Number of compounds per sample among *Cypripedium calceolus* (circles) and six co-flowering rewarding species (diamonds). Each symbol represents an individual. Boxplots are shown for each species and indicate the median, the first and third quartiles, and maximum and minimum values. Individuals and boxplots are color-coded according to species. Different lowercase letters at the top of the graph indicate statistically significant differences in the number of scent compounds recorded (Dunn’s test).

In total, 105 scent compounds belonging to seven compound classes were detected across all samples of *C. calceolus* and the six co-flowering species (**Table 1**; **Supplementary Table S2**). The number of compounds per scent sample differed among species (Kruskal–Wallis rank sum test: *χ* ^2^_6_ = 38.05, p < 0.001) with *C. calceolus* emitting significantly more compounds than all other species except *H. comosa* (**Figure 2**). Across all *C. calceolus* samples, 67 compounds belonging to six compound classes were detected—20 aliphatic compounds, 12 aromatic compounds, 24 terpenoids, one C5-branched chain compound, one nitrogen-containing compound, and nine unknown compounds—with the two aliphatic compounds octyl acetate (mean relative amount: 32%) and decyl acetate (8%) and the terpenoid linalool (30%) having a mean relative amount of at least 5% (**Table 1**). These were more scent compounds than we detected across all samples of the six co-flowering species together (58 compounds) (**Table 1**). The number of compounds emitted in the co-flowering species ranged from 2 to 23 (**Table 1**). The samples of *L. incanus* and *G. cordifolia* contained only aromatic compounds, two in *L. incanus* (benzaldehyde with a mean relative amount of 69%, benzyl alcohol: 31%) and three in *G. cordifolia* (1,2-dimethoxybenzene: 77%, eugenol: 17%, and guaiacol: 5%) (**Table 1**). The samples of *D. octopetala* comprised 11 compounds, 9 terpenoids, and 2 unknown compounds, and were dominated by 4 isomers of lilac aldehyde, together accounting for 53% of the total scent emission, followed by lavender lactone (24%) and (*Z*)-arbusculone (9%) (**Table 1**). The samples of *P. farinosa* and *H. bifidum* comprised aromatic compounds, terpenoids, and unknown compounds (**Table 1**). Nine compounds were detected in the samples of *P. farinosa*, mainly 4-oxoisophorone (60%), *p*-cresol (18%), and benzyl alcohol (16%), and the samples of *H. bifidum* contained 14 compounds among which benzaldehyde (58%) and benzyl alcohol (25%) were the dominant compounds (**Table 1**). The samples of *H. comosa* contained 23 compounds belonging to five compound classes (aromatic compounds, terpenoids, nitrogen-containing compounds, miscellaneous cyclic compounds, and unknown compounds), with the scent being dominated by methyl-2-hydroxy-3-phenylpropionate (27%), an unknown compound (23%), and indole (18%).

**Figure 3:**
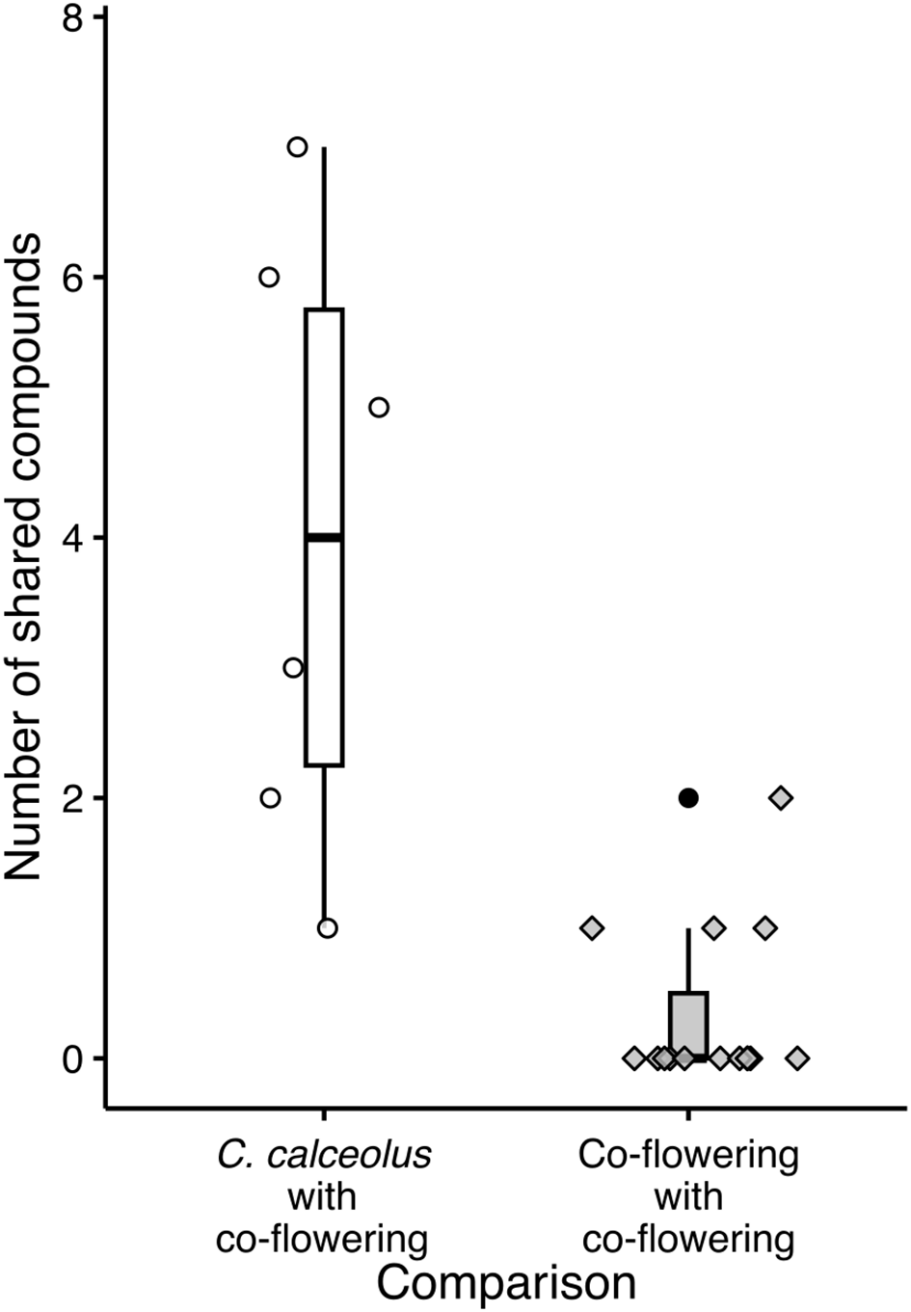
Number of shared compounds between *Cypripedium calceolus* with the six co-flowering rewarding species (“*C. calceolus* with co-flowering”) and the six co-flowering rewarding species with each other (“Co-flowering with co-flowering”). Each symbol represents a pairwise species comparison. Boxplots indicate the median, the first and third quartiles, and maximum and minimum values.

**Figure 4:**
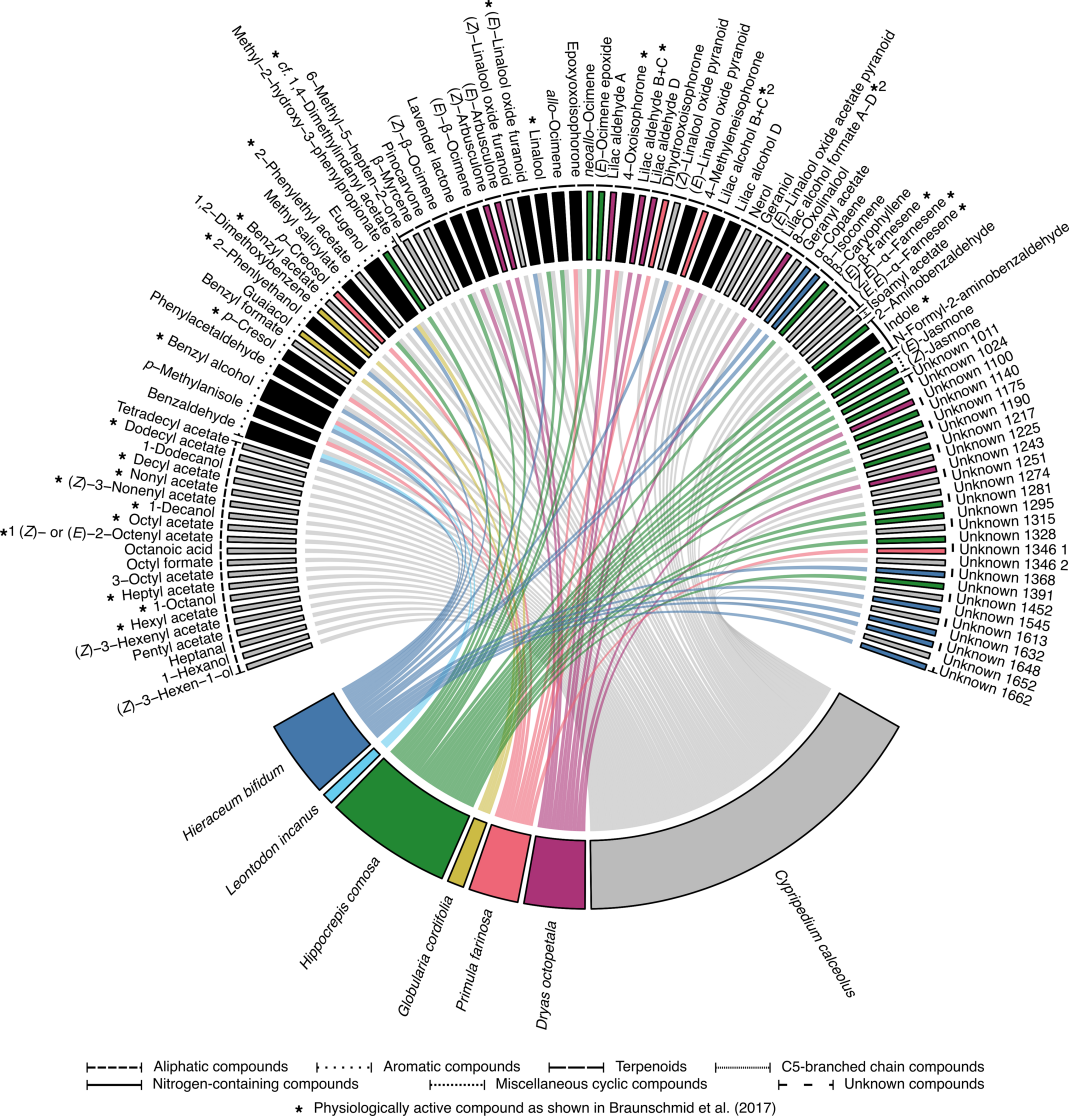
Chord diagram showing *Cypripedium calceolus* and the six co-flowering rewarding species as well as each floral scent compound by a separate segment. The ribbons link the species to the scent compounds. Species and species-specific compounds are color-coded, whereas compounds shared between *C. calceolus* and one or more co-flowering species are given in black. Compounds are grouped by compound classes, which are highlighted by different line styles between the compound name and the compound segment as indicated at the bottom of the figure. Compounds that have been shown to be physiologically active in antennae of pollinators of *C. calceolus* in a previous study by Braunschmid et al. (2017) are indicated by “*”. *^1^EAD activity has been shown for the stereoisomer (*E*)-2-octenly acetate (Braunschmid et al., 2017), *^2^EAD activity has been indicated for pooled A–C stereoisomers without discriminating among the single stereoisomers by Braunschmid et al. (2017).

Overall, *C. calceolus* shared, with a median of 3.5 scent compounds, significantly more compounds with the co-flowering rewarding plants than the co-flowering plant species shared among each other (median 0; Mann–Whitney U-test: *Z*_n1 = 6, n2 = 15_ = 3.31, p < 0.001; **Figure 3**). Not a single floral scent compound was found occurring in all of the six co-flowering species, and only three substances were present in more than one species: benzyl alcohol was found in three species (*H. bifidum*, *L. incanus* and *P. farinosa*), benzaldehyde in two species (*H. bifidum* and *L. incanus*), and eugenol in two species (*H. bifidum* and *G. cordifolia*). *Cypripedium calceolus*, in contrast, shared 20 floral scent compounds (30% of the total of 67 compounds that were found across all *C. calceolus* samples) with the co-flowering community (**Table 1**; **Figure 4**). Whereas most of them were shared with only one other species, the scent compound benzyl alcohol was shared with three and the compounds benzaldehyde and eugenol with two co-flowering species. Overall, *C. calceolus* shared seven compounds (the most compounds) with *H. bifidum*, six compounds each with *H. comosa* and *P. farinosa*, two compounds each with *D. octopetala* and *L. incanus*, and one compound with *G. cordifolia*. The shared compounds all belonged to the class of aromatic compounds, terpenoids, and nitrogen-containing compounds. Interestingly, 20 of the 67 compounds in *C. calceolus* were aliphatic compounds, but no aliphatic compounds were detected in the co-flowering plant species studied.

One of the main compounds of *C. calceolus*, linalool (30%), was shared with *H. comosa* in which it contributed 1% to the floral bouquet. Contrary to that, eight other compounds, which occur only in small amounts (<4%) in *C. calceolus*, were found to be among the main compounds in another species: 4-oxoisophorone in *P. farinosa* (60%); benzaldehyde in *L. incanus* (55%) and *H. bifidum* (58%); benzyl alcohol in *H. bifidum* (25%), *L. incanus* (25%), and *P. farinosa* (16%); lavender lactone in *D. octopetala* (24%); indole in *H. comosa* (18%); *p*-cresol in *P. farinosa* (18%); eugenol in *G. cordifolia* (17%); and (*E*)-β-ocimene in *H. comosa* (8%). Other shared substances, which occurred both in *C. calceolus* and in its co-flowering species only in small amounts (≤5%), were 2-phenylethanol, phenylacetaldehyde, eugenol, (*E*)-linalool oxide furanoid, and (*E*)-linalool oxide pyranoid for *H. bifidum*; methyl salicylate, (*Z*)-β-ocimene, and *allo*-ocimene for *H. comosa*; lilac alcohol B+C for *D. octopetala*; and epoxyoxoisophorone, *p*-methylanisole, and one unknown substance for *P. farinosa*.

**Figure 5:**
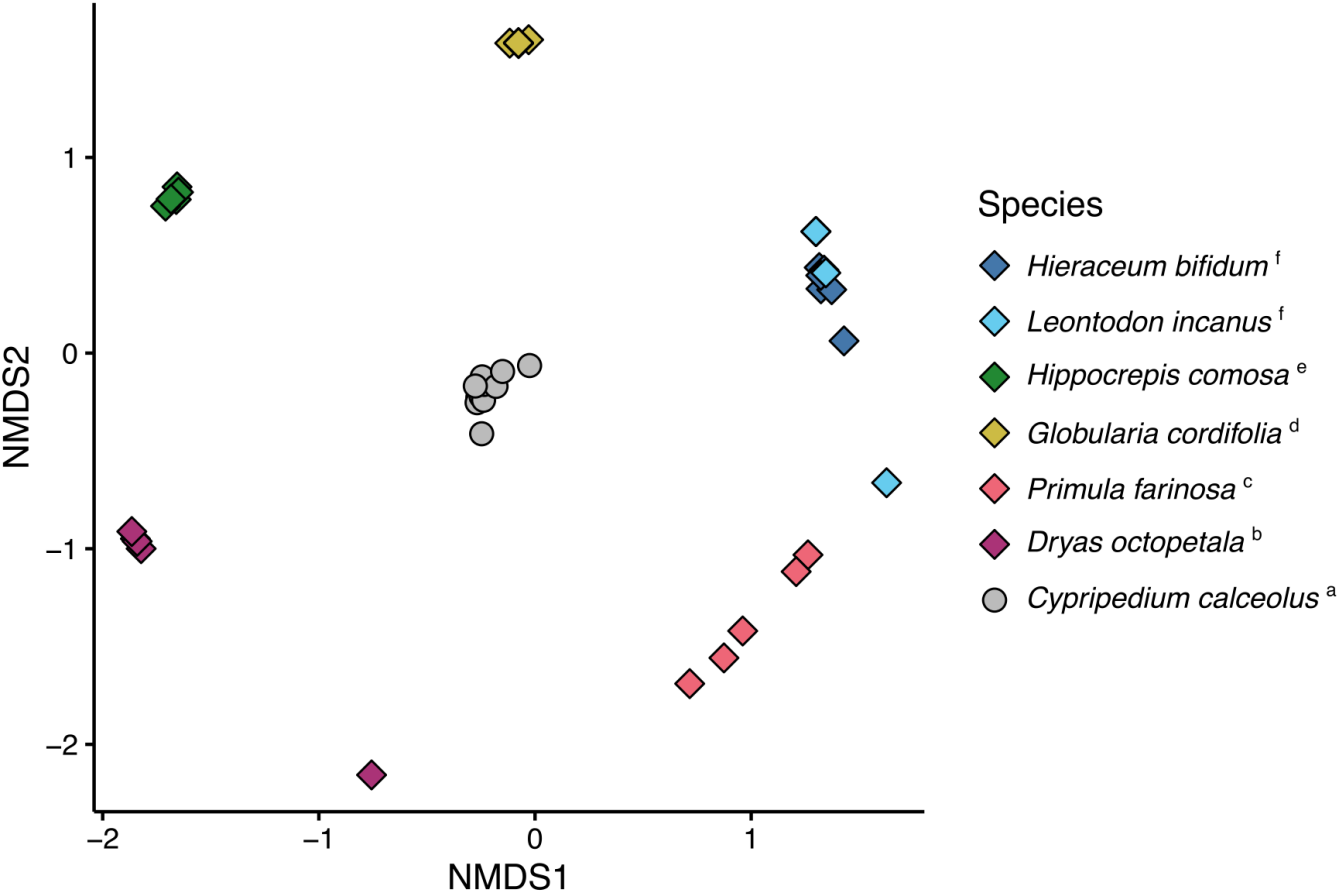
Non-metric multidimensional scaling (NMDS), based on pairwise Bray–Curtis similarities, used to visualize semi-quantitative (dis)similarities among floral bouquets of *Cypripedium calceolus* (circles) and six co-flowering rewarding species (diamonds). Each symbol represents an individual, and individuals are color-coded according to species. The 2D stress of the NMDS was 0.04. Different lowercase letters at the end of the species names indicate statistically significant differences in scent composition (pairwise PERMANOVA).

At the semiquantitative level, too, floral scent differed among species (PERMANOVA: pseudo-*F*_6,38_ = 25.91, *p* < 0.001) with all pairwise species comparisons being statistically significant except for *H. bifidum* and *L. incanus* (**Figure 5**). Multivariate dispersion of floral scent, however, did not differ among species (PERMDISP: *F*_6,38_ = 2.10, p = 0.290). The scent of *C. calceolus* showed a relatively unique scent profile, but at the same time shared numerous floral compounds with its co-flowering plant community, so that, based on the dissimilarities of the floral scent bouquets, all *C. calceolus* individuals grouped in the center, and the co-flowering species spread in a circular pattern around *C. calceolus* and grouped according to species, except *L. incanus* and *H. bifidum*, which partially overlapped with each other (**Figure 5**).

## 4 Discussion

Our study in the food-deceptive orchid *C. calceolus* on floral scent imitation of the co-flowering rewarding plant community showed that *C. calceolus* shared scent compounds with all co-flowering species that emitted detectable floral scent and that (potentially) share pollinators with *C. calceolus*. Almost one-third of the 67 floral scent compounds of *C. calceolus* were also found in at least one of the co-flowering species, among them compounds generally widespread among floral scents but also less widespread compounds (Knudsen et al., 2006). Eight of the shared compounds have previously been shown in physiological measurements to elicit an antennal response in bees and hoverflies that pollinate *C. calceolus* (Braunschmid et al., 2017). Moreover, *C. calceolus* shared more compounds with the co-flowering rewarding plant community than the co-flowering species shared among each other. Together, these results indicate that (food-)deceptive orchids may not only emit compounds that are common among flowering plants and attractive to many pollinators but also specifically mimic floral scents of multiple co-flowering plant species.

Many food-deceptive orchids emit floral scent (e.g., in *Cephalanthera rubra*: Nilsson, 1983; *Anacamptis morio*: Nilsson, 1984; *Epidendrum ciliare*: Moya and Ackerman, 1993; *Tolumnia variegata*: Ackerman et al., 1997; *Orchis mascula*, *O. pauciflora*, and their hybrid *O. × colemanii*: Salzmann et al., 2007a; *Dactylorhiza romana*: Salzmann and Schiestl, 2007; and *Traunsteineria globosa*: Jersáková et al., 2016), with 11–49 compounds per species. Interestingly, with 67 compounds in our study population, *C. calceolus* emits more than these generalized food-deceptive orchids. This indicates that floral scent of *C. calceolus* might have some additional functions, by imitating specific models, than floral scent has in other food-deceptive species.

Our assessment of the floral scent of co-flowering rewarding species of *C. calceolus* helps to better understand such additional functions. For the four co-flowering species, *L. incanus*, *H. comosa*, *G. cordifolia*, and *D. octopetala*, floral scent has, to our knowledge, not been described before. For the remaining two co-flowering rewarding species, floral scent has previously been studied, and similar floral scent compounds to the ones we encountered had been found, but some compounds were different (*P. farinosa*: Gaskett et al., 2005; *H. bifidum*: Feulner et al., 2011). Such differences could partially arise through the usage of (slightly) different scent collection and analysis methods but could also reflect phenotypic plasticity (e.g., Majetic et al., 2009) or be the result of geographical differences in selection, for example, imposed by pollinators as suggested in other plant species (e.g., Gross et al., 2016; Chapurlat et al., 2018).

Two recent studies in food-deceptive orchids have compared the scent profile, as well as other floral traits, to co-flowering rewarding species (Jersáková et al., 2006; Scaccabarozzi et al., 2025). *Traunsteineria globosa* has been found to share almost 70% of its floral scent compounds with co-flowering rewarding species of *Knautia* and *Scabiosa* (Dipsacaceae) and *Valeriana* (Caprifoliaceae) to which it closely resembles in floral color and in the compact inflorescences (Jersáková et al., 2006). As fly pollinators did not discriminate between *T. globosa* and its potential model species but bees and butterflies did, Jersáková et al. (2006) suggested that *T. globosa* quite closely mimics its potential model species in visual signals but that bees and butterflies are able to discriminate the deceptive orchid from the rewarding species based on the differences in scent. In two species of the orchid genus *Thelymitra*, the floral scent of these orchids is quite similar to that of one species each of the rewarding tinsel lilies (*Calectasia* spp.) that have been suggested to be model species because of high similarities in flower color and especially flower morphology. However, the scent of the orchids is also quite similar to that of other co-flowering rewarding species that differ in flower color and morphology from the *Thelymitra* species (Scaccabarozzi et al., 2025). In this system, it has been suggested that floral scent may reinforce the similarity in floral color and morphology (Scaccabarozzi et al., 2025). In contrast, *C. calceolus* strongly differed morphologically from co-flowering rewarding species, and the yellow color of the prominent lip of *C. calceolus* was, to the human eye, similar only to that of some of the co-flowering rewarding species in our study population (yellow flowers of *H. bifidum*, *L. incanus*, and *H. comosa* and the yellow center of the flowers of *D. octopetala*). The floral scent of *C. calceolus*, too, differed qualitatively and quantitatively from that of the six co-flowering rewarding species. Nevertheless, *C. calceolus* shared 30% of its compounds and at least one and up to seven compounds with each of its co-flowering rewarding species. Thus, sharing some compounds with a variety of co-flowering rewarding species might be advantageous for *C. calceolus* in attracting pollinators.

Generalized food deception and Batesian food source mimicry probably represent two extremes of a continuum, and Batesian food source mimicry is thought to have evolved from generalized food deception (Jersáková et al., 2009; Qu et al., 2023; D’Aria et al., 2024). In our study, we find support for both strategies. In support of a generalized food-deceptive pollination strategy is our finding that the floral scent of *C. calceolus* contained 8 [(*E*)-β-ocimene, β-myrcene, linalool, benzaldehyde, methyl salicylate, benzyl alcohol, 2-phenylethanol, 6-methyl-5-hepten-2-one] of the 12 compounds that have been identified to occur in more than half of the seed plant families (Knudsen et al., 2006) and shared all of these except two of them (myrcene, 6-methyl-5-hepten-2-one) with at least one co-flowering species. Other generalized food-deceptive plants (e.g., *Dactylorhiza sambuccina*, *Anacamptis morio* and *A. pyramidalis*, *Caladenia longicauda*, *Orchis mascula* and *O. pauciflora*, and *Traunsteinera globosa*) contain 4 to 9 of these 12 most widespread compounds, which is 12%–45% of their compounds, and the 8 widespread compounds in the floral scent of *C. calceolus* in our study population are also found in the floral scent of at least one of these other food-deceptive species (Nilsson, 1980, 1984; Andersson et al., 2002; Salzmann et al., 2006, 2007b, 2007a; Jersáková et al., 2016). In comparison, *C. calceolus* contains a relatively low proportion (12%) of the 12 most widespread compounds and, thus, might have a broader mimicry strategy than these other generalized food-deceptive species. Indeed, we also found support for more specific imitation of the floral scent of co-flowering rewarding species in *C. calceolus. Cypripedium calceolus* did not only overlap in six of the most widespread compounds with the co-flowering rewarding species in our study population but also in 14 other, rarer compounds. Interestingly, three of these compounds (*p*-cresol, indole, epoxyoxoisophorone) are exclusively found in samples of the northern Alps but not south of the Alps or in Scandinavia (Braunschmid et al., 2021), and two of these compounds (*p*-cresol, indole) are physiologically active in solitary bees and hoverflies (Braunschmid et al., 2017) and, thus, might reflect an adaptation to local differences in the pollinator and/or the co-flowering rewarding plant community. However, it has not yet been tested which of the physiologically active floral scent compounds in *C. calceolus* are involved in pollinator attraction, but several compounds have been shown to attract insect species of the same genus or closely related genera as the pollinators of *C. calceolus*. For example, benzaldehyde, 4-oxoisophorone, and the combination thereof attracts several hoverfly species and one *Lasioglossum* species (El-Sayed et al., 2018); eugenol attracts the hoverfly *Eupeodes corollae* (Li et al., 2020); β-ocimene attracts honeybees (Pecetti et al., 2002); compounds, such as phenylacetaldehyde, methyl salicylate, linalool oxide pyranoid, linalool, and 2-phenylethanol, attract hoverflies (Primante and Dötterl, 2010; reviewed in Dötterl and Gershenzon, 2023 and references therein); *p*-anisaldehyde and phenylacetaldehyde attracts Halictidae (e.g., *Lasioglossum*) (Meagher, 2002; Theis, 2006); and (*E*,*E*)-α-farnesene, linalool, methyl salicylate, and 2-phenylethanol attract *Andrena vaga* (which is, however, not a pollinator of *C. calceolus*) (Dötterl and Vereecken, 2010 and references therein). Thus, several of the floral scent compounds of *C. calceolus*, including some that *C. calceolus* shared with the co-flowering rewarding species, might indeed affect the behavior of *C. calceolus* pollinators and lure them to the rewardless flowers. Together, these findings indicate that *C. calceolus* may have a pollination strategy between generalized food deception and Batesian floral mimicry. It is probably closer to generalized food deception than the pollination strategy of *T. globosa*, which has been suggested to have a guild mimicry strategy, and the pollination strategy of the *Thelymitra* system, which is closest to Batesian food source mimicry.

Interestingly, *C. calceolus* in our study population contained 20 aliphatic compounds, two of which (octyl acetate, decyl acetate) constituted more than 5% of the total scent. Aliphatic compounds, in general, and those in the scent bouquet of *C. calceolus*, in particular, are neither among the most widespread floral scent compounds (see Knudsen et al., 2006) nor among the compounds we detected in the co-flowering rewarding species. Several of these compounds, however, are well known from species-specific cephalic secretions of female and male *Andrena* bees (Tengö and Bergström, 1977; El-Sayed, 2025). For example, cephalic secretions of *Andrena haemorrhoa*, *A. jacobi* (=*A. carantonica*), and *A. nigroaenea*, all known as pollinators of *C. calceolus* (Braunschmid et al., 2021), have, among others, 1-dodecanol (*A. haemorrhoa*, *A. jacobi*), decyl, dodecyl and tetradecyl acetate (*A. haemorrhoa*), 1-octanol and 1-decanol (*A. jacobi*), and octyl acetate (*A. nigroaenea*) in common with floral scents of the studied *C. calceolus* population. These compounds are male aggregation and sex pheromones of *Andrena* spp. and were already previously discussed as being potentially involved in the attraction of *Andrena* pollinators to *C. calceolus* (Nilsson, 1979). In the studied population of *C. calceolus*, as is true for other populations in the northern Alps, however, *Andrena* bees are only minor pollinators (Braunschmid et al., 2017, 2021), and from the main *Lasioglossum* pollinators, such aliphatic compounds are not known to occur as pheromones (El-Sayed, 2025).

Overall, our results indicate that *C. calceolus* has a pollination strategy intermediate between generalized food deception, Batesian floral mimicry, and Batesian pheromone mimicry, and this might explain why this species emits more compounds than other food-deceptive plants. While such a triple deceptive strategy seems to be specific for *C. calceolus*, intermediate pollination strategies between generalized food deception and Batesian floral mimicry have also been suggested for other food-deceptive orchids (Jersáková et al., 2016; D’Aria et al., 2024). The pollination strategy of *C. calceolus* might reflect an evolutionary transition from generalized food deception to Batesian floral mimicry. Alternatively, it could reflect an adaptation to the exposure to a variable pollinator assemblage across the wide distribution range of *C. calceolus*, and thus, depending on the locally available pollinators and co-flowering rewarding plants, the relative importance of the different deceptive strategies might differ among populations. The majority of the species of the genus *Cypripedium* have a food-deceptive strategy, but other pollination strategies have been suggested. For example, generalized food deception has been proposed in *C. guttatum*, where the similarities with co-flowering species in color are suggested to be the results of a diverse co-occurring flora rather than floral mimicry (Bänziger et al., 2005). Several other species are highly specialized in mimicking, for example, fly oviposition sites (Ren et al., 2011; Li et al., 2012). Another example is the mimicry of a specific food-rewarding model plant (*Pedicularis schistostegia*, Orobanchaceae), which has, so far, only been suggested for *C. macranthos* var. *rebunense* (Sugiura et al., 2001, 2002). Thus, *C. calceolus* and other congeners might be an interesting system to study the evolutionary transition from generalized food deception to specialized deception pollination strategies.

## Data Availability

The original contributions presented in the study are included in the article/**Supplementary Material**. Further inquiries can be directed to the corresponding author.

## Supplementary material

Supplementary Table 1 Total absolute amount of scent trapped [ng/h per pollination unit] separately for each sample of the six co-flowering rewarding species.

Supplementary Data Sheet 1 Relative amount [%] for all 105 floral scent compounds and the number of compounds emitted separately for each sample of the six co-flowering rewarding species and *Cypripedium calceolus*.
