# Supplementary material for: Deceptive Cypripedium calceolus shares more floral scent compounds with co-flowering rewarding species than those species share among each other
Source: Front Plant Sci. 2025 Aug 12;16:1627890. doi: 10.3389/fpls.2025.1627890 (PMC12378319; doi:10.3389/fpls.2025.1627890)
Supplement: Supplementary Data Sheet 1 — Relative amount [%] for all 105 floral scent compounds and the number of compounds emitted separately for each sample of the six co-flowering rewarding species and Cypripedium calceolus. [file DataSheet1.pdf]

Supplementary Material

Food-deceptive *Cypripedium calceolus* shares more floral scent compounds with co-flowering rewarding species than those among each other

Corinna Etl, Florian Etl, Robin Guilhot, Herbert Braunschmid, Karin Gross, Stefan Dötterl

The following Supplementary Material is available for this article:

Table S1 Total absolute amount of scent for each sample.

Table S2 Relative amount of all 105 scent compounds and the number of scent compounds emitted for each sample. (Note: Is a separate Excel file.)

Table S1 Total absolute amount of scent trapped [ng/h per pollination unit] separately for each sample of the six co-flowering rewarding species.

Plant ID	Species	Total amount [ng/h per pollination unit]
3Dry	*Dryas octopetala*	39
4Dry	*Dryas octopetala*	18
5Dry	*Dryas octopetala*	38
6Dry	*Dryas octopetala*	1
7Dry	*Dryas octopetala*	81
10Hie	*Hieraceum bifidum*	169
34Hie	*Hieraceum bifidum*	52
39Hie	*Hieraceum bifidum*	125
40Hie	*Hieraceum bifidum*	191
41Hie	*Hieraceum bifidum*	134
R32Hie	*Hieraceum bifidum*	544
R34Hie	*Hieraceum bifidum*	92
15Leo	*Leontodon incanus*	22
21Leo	*Leontodon incanus*	31
23Leo	*Leontodon incanus*	20
27Leo	*Leontodon incanus*	14
12Hip	*Hippocrepis comosa*	2191
18Hip	*Hippocrepis comosa*	224
26Hip	*Hippocrepis comosa*	1486
35Hip	*Hippocrepis comosa*	1769
38Hip	*Hippocrepis comosa*	1998
52Glo	*Globularia cordifolia*	14
54Glo	*Globularia cordifolia*	19
R54Glo	*Globularia cordifolia*	124
R75Glo	*Globularia cordifolia*	100
R83Glo	*Globularia cordifolia*	56
76Pri	*Primula farinosa*	31
77Pri	*Primula farinosa*	206
78Pri	*Primula farinosa*	672
79Pri	*Primula farinosa*	80
45Pri	*Primula farinosa*	120
